# Transcriptional Downregulation of Rice rpL32 Gene under Abiotic Stress Is Associated with Removal of Transcription Factors within the Promoter Region

**DOI:** 10.1371/journal.pone.0028058

**Published:** 2011-11-23

**Authors:** Pradipto Mukhopadhyay, Malireddy K. Reddy, Sneh Lata Singla-Pareek, Sudhir K. Sopory

**Affiliations:** Plant Molecular Biology Group, International Centre for Genetic Engineering and Biotechnology, New Delhi, Delhi, India; University of Louisville, United States of America

## Abstract

**Background:**

The regulation of ribosomal proteins in plants under stress conditions has not been well studied. Although a few reports have shown stress-specific post-transcriptional and translational mechanisms involved in downregulation of ribosomal proteins yet stress-responsive transcriptional regulation of ribosomal proteins is largely unknown in plants.

**Methodology/Principal Findings:**

In the present work, transcriptional regulation of genes encoding rice 60S ribosomal protein L32 (rpL32) in response to salt stress has been studied. Northern and RT-PCR analyses showed a significant downregulation of *rpL32* transcripts under abiotic stress conditions in rice. Of the four *rpL32* genes in rice genome, the gene on chromosome 8 (*rpL32_8.1*) showed a higher degree of stress-responsive downregulation in salt sensitive rice variety than in tolerant one and its expression reverted to its original level upon withdrawal of stress. The nuclear run-on and promoter:reporter assays revealed that the downregulation of this gene is transcriptional and originates within the promoter region. Using *in vivo* footprinting and electrophoretic mobility shift assay (EMSA), *cis*-elements in the promoter of *rpL32_8.1* showing reduced binding to proteins in shoots of salt stressed rice seedlings were identified.

**Conclusions:**

The present work is one of the few reports on study of stress downregulated genes. The data revealed that *rpL32* gene is transcriptionally downregulated under abiotic stress in rice and that this transcriptional downregulation is associated with the removal of transcription factors from specific promoter elements.

## Introduction

Plants encounter a number of abiotic stresses which limit agricultural production. Classical breeding programs, as also the initial attempts with genetic engineering to raise stress tolerant plants have met with limited success [1]. Through recent advances in genomics and proteomics, some knowledge about stress perception, stress signaling and transcription regulators has been gained from model organisms such as *Arabidopsis, Synechocystis* and *Clamydomonas* [2]–[4]. Yet a more detailed understanding of stress response and adaptation is desirable, especially for monocot crop plants like rice [5]. An in depth knowledge about the regulation of stress-responsive genes will help in developing strategies to increase the stress tolerance level of socially and economically important crops.

It is well known that growth is severely affected in plants under stress conditions. Ribosomal proteins are essential for proper growth and development of any organism. Not much was known about regulation of expression of genes encoding ribosomal proteins in plants until recently. Many ribosomal proteins have been reported to be up- or downregulated under various stress conditions [6]–[10]. Recently, chloroplast encoded rpL33 was shown to be necessary for plant survival under cold stress in *Arabidopsis* [11]. Pathways of translational downregulation of ribosomal proteins as observed in mammals and transcriptional regulation of ribosomal proteins, somewhat similar to yeast, have been described in plants earlier [12]–[17]. Post-transcriptional regulation for ribosomal proteins has also been suggested [7], [18]–[21]. Although altered expression of ribosomal proteins under stress has been shown to involve the post-transcriptional and translational regulatory mechanisms, stress-specific transcriptional regulation of ribosomal proteins is still to be reported in plants.

In this work, we have studied the transcriptional regulation of genes encoding 60S ribosomal protein rpL32 in rice under salt stress. Homologues of rpL32 have been shown to bind RNA in archaea and maize, possess transcriptional transactivation activity, mediate telomere silencing in yeast, and promote proper growth and development in *Drosophila* [22]–[25]. From the analysis of differential cDNA libraries of rice (previous work in our lab; unpublished) and barley [26], the downregulation of this gene under salt stress was postulated. The present work has shown that *rpL32* gene is downregulated at the transcriptional level under abiotic stress and that this downregulation is linked to the removal of proteins from *cis-*elements in the promoter region. Our work presents a study of transcriptional downregulation of genes which has been far less studied than upregulation of genes during abiotic stress in plants.

## Results

### Rice genome contain four genes encoding ribosomal protein rpL32

A BLASTn homology search using partial coding sequence of rpL32 in the rice genome database of NCBI (http://www.ncbi.nlm.nih.gov/) and MSU Rice Genome Annotation Project v6.1 (RGAP 6.1; http://rice.plantbiology.msu.edu/) indicated the presence of four *rpL32* genes (Fig. S1). These loci have been annotated as putative 60S *rpL32* gene in the NCBI database. One of these genes was present on chromosome 8 and was named *rpL32_8.1*. The other three were named *rpL32_9.1*, *rpL32_9.2*, and *rpL32_9.3* and located contiguously on chromosome 9. The CDS and the predicted protein of all the four rpL32 members share a high percentage of identity (Fig. S2A,B). However, the 3′ UTR sequence of these genes were dissimilar (Fig. S3). BLASTn homology search using 3′UTR specific probes for each of the predicted *rpL32* genes in NCBI database, revealed the presence of homologous expressed sequence tags (ESTs) and mRNA sequences. This confirmed that all these four genes are expressed in rice. The gene structure is also similar for all the *rpL32* genes of rice, each having three introns (Fig. S2C). The first intron is the smallest one and resides within the 5′ UTR of the genes. The location of the remaining introns, on the other hand differs in each gene.

### Rice *rpL32* genes are downregulated in shoots under salt stress

**Figure 1:**
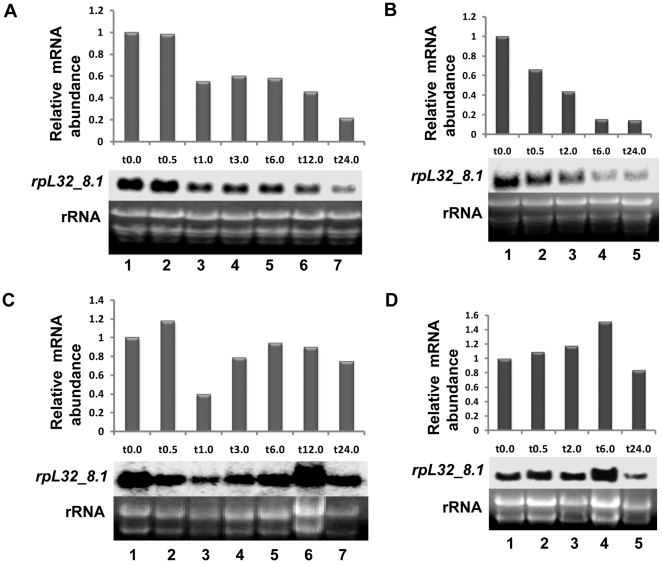
Northern blot analysis of *rpL32_8.1* under salt stress. (A) Pokkali shoot (B) PB1 shoot (C) Pokkali root (D) PB1 root. t0.0 to t24.0 indicate the time of treatment (in h) with 200 mM NaCl ranging from 0 h to 24 h. The graphical representation of the data was deduced by normalizing the densitometric intensity of *rpL32* genes on the blot with that of 28S rRNA in the corresponding lane of the EtBr gel.

Northern hybridization using radiolabeled 3′UTR probe of *rpL32_8.1* revealed that this gene is highly expressed under control (unstressed) conditions in shoots of salt tolerant indica rice Pokkali (Fig. 1A). After 1 to 12 h of salt stress, a 40–60% decrease in steady-state level of the transcript was observed relative to the unstressed condition (t0.0).The level of *rpL32_8.1* decreased further to 20% of control after 24 h of salt treatment. In the salt sensitive PB1 variety of indica rice also, the level of *rpL32_8.1* decreased in response to salt stress except that the response was more rapid and of greater magnitude than that observed in the salt tolerant pokkali variety (Fig. 1B). The root tissues of Pokkali and PB1 rice seedlings did not exhibit any steady decrease in their transcript level (Fig. 1C,D).

**Figure 2:**
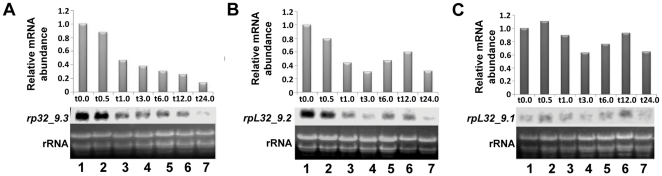
Northern blot analysis of *rpL32* genes located on chromosome 9 under salt stress. (A) *rpL32_9.3,* (B) *rpL32_9.2,* (C) *rpL32_9.1*. t0.0 to t24.0 indicate the time after treatment of the Pokkali rice seedlings with 200 mM NaCl ranging from 0 to 24 h. All analyses were done as mentioned for Figure 1.

*rpL32_9.3* and *rpL32_9.2* also exhibited downregulation of their expression in response to salt stress in shoots of Pokkali rice (Fig. 2A,B). However, the decrease in transcript level of *rpL32_9.1* was relatively less under salt stress than the other three *rpL32* genes (Fig. 2C). The transcript of this gene was also faintly detected in northern blots as compared to other *rpL32* genes.

A semiquantitative RT-PCR, using *elongation factor-1α* (*EF-1α*) as internal control gene, reconfirmed the differential downregulation of *rpL32* genes in shoots of PB1 and Pokkali rice plants under salt stress (Fig. S4A–C). Though small differences in the degree of downregulation of these genes by RT-PCR and northern analyses were observed, yet the faster rate of shoot-specific downregulation of *rpL32_8.1* under salt stress in PB1 rice variety was validated. Using RT-PCR, downregulation of *rpL32_9.1* under salt stress was also verified.

### Downregulation of *rpL32* genes under stress is transcriptional in origin

**Figure 3:**
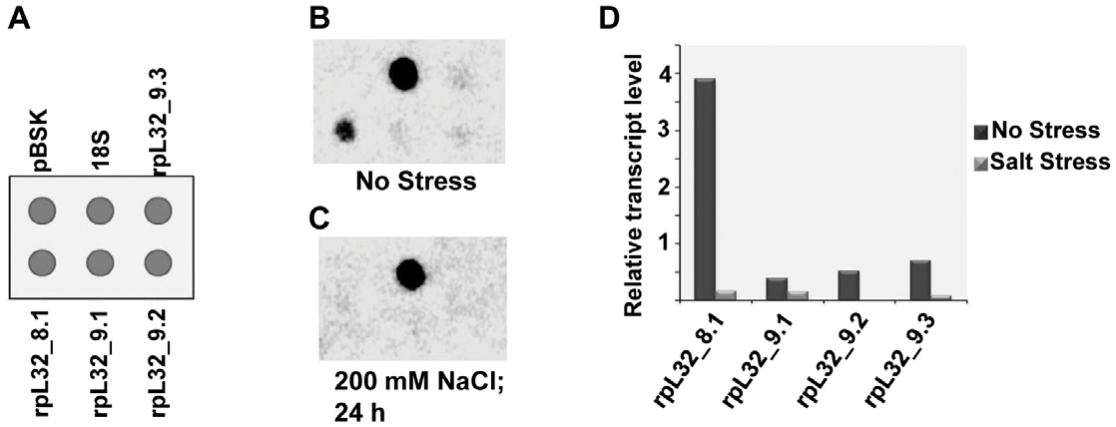
Nuclear Run-on analysis depicting the transcriptional activity of the *rpL32* genes in shoots. (A) Pattern of loading of different genes in the dot blots. The blots were hybridized with 3.45×10^6^ cpm/ml probe count. (B) Blot for control (no stress) condition. (C) Blot for salt stress (200 mM NaCl, 24 h) condition. (D) Graphical representation of the results of nuclear run-on assay. Densitometric quantification values for *rpL32* genes were normalized with the densitometric readings obtained for 18S rRNA.

To determine if the downregulation of *rpL32* genes happens at transcriptional level, nuclear run-on assay was performed using nuclei isolated from unstressed and stressed shoots (Fig. 3). Nuclear run-on assay of nuclei from unstressed shoots revealed that all four *rpL32* genes in rice were expressed, with the strongest transcription being of *rpL32_8.1* (nearly 8–10 folds higher than the other *rpL32* genes; Fig. 3B,D). Although such differences might result from different hybridization efficiencies of the 3′UTR specific DNA fragments used in the dot blot for the detection of *rpL32* genes, it appeared least likely as these 3′UTR regions are similar in length and GC content. Moreover, during RT-PCR analysis also, *rpL32_8.1* was detected with lesser number of PCR cycles (than that used for other *rpL32* genes). Transcription was greatly reduced in stressed shoots (treated with 200 mM NaCl for 24 h), with the signal for all the *rpL32* genes being barely visible (Fig. 3C,D). This indicated that these genes are transcriptionally downregulated. As the results of this assay indicated that the majority of the *rpL32* transcript in rice is contributed by *rpL32_8.1*, further studies were focused on the regulation of this gene.

### *rpL32_8.1* gene expression is responsive to various treatments

**Figure 4:**
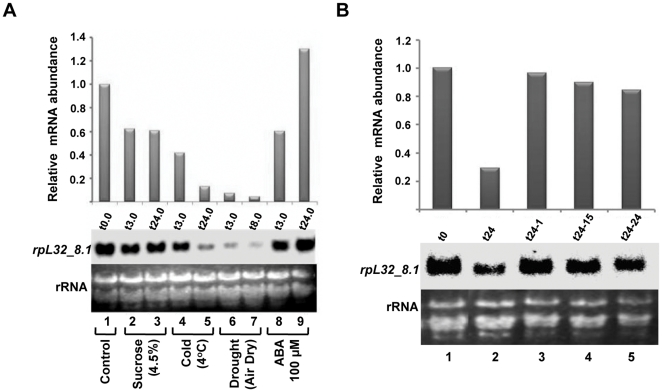
Northern blot analysis of *rpL32_8.1* under various treatments and during stress recovery. (A) Expression analysis under various treatments (sucrose, cold, drought and ABA) as mentioned below each lane. t0.0 to t24.0 indicate the time of treatment (in h). (B) Transcript abundance during stress recovery. t0 (lane 1) and t24 (lane 2) indicate the control (unstressed) and 24 h stressed (200 mM NaCl) tissues. t24-1 (lane 3)**,** t24-15 (lane 4) and t24-24 (lane 5) indicate 1, 15 and 24 h of incubation during recovery from salt stress. All analyses were done as mentioned for Figure 1.

To investigate if *rpL32_8.1* gene is responsive to other factors, its expression was studied under a variety of conditions (Fig. 4A). As compared to untreated samples, about 40% and 60–85% reduction in the transcript level was observed in response to sucrose (4.5%) and cold (4°C) treatments (for 3 and 24 h), respectively. With more than 90% reduction in the transcript level by 3 h, the most drastic effect on *rpL32_8.1* expression was observed in response to drought stress (air-drying). Although some reduction in the transcript level was evident after 3 h of abscisic acid (ABA; 100 µM) treatment, such downregulation was not observed after 24 h.

The transcript level of *rpL32_8.1* gene was also measured in the shoots of seedlings during stress recovery (Fig. 4B). The seedlings were given 200 mM NaCl stress for 24 h, after which they were placed back onto the medium lacking NaCl. After 24 h of salt stress, expression of *rpL32_8.1* was reduced as observed earlier (Fig. 4B; Lane2). However, within 1 h of stress recovery, the transcript level increased and reached the levels found in unstressed shoots (Fig. 4B; Lane 3–5). This indicates that the regulation of this gene is dynamic and reversible, and the gene is downregulated only till such time as the plants are exposed to stress.

Semiquantitative RT-PCR analysis reconfirmed the decline of this transcript under cold and drought stress, and its increase during recovery from NaCl stress. (Fig. S4D–F). However, ABA treatment did not show any significant downregulation of *rpL32_8.1* expression. RT-PCR showed a smaller decrease (20–30%) in transcript levels under sucrose treatment than that observed by northern analysis. Similar to northern studies, this method also indicated that the extent of downregulation of *rpL32_8.1* expression was greater in drought stress than under salt and cold stress.

### The transcriptional regulation of *rpL32_8.1* is governed by its promoter

**Figure 5:**
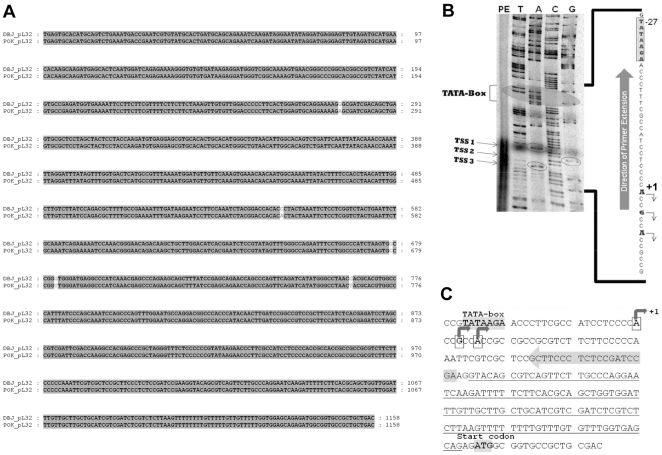
Promoter sequence analysis and transcription start site (TSS) mapping of *rpL32_8.1.* (A) A ClustalW alignment of the japonica rice and indica rice Pokkali sequences corresponding to isolated genomic DNA portion. (B) Autoradiogram of denaturing urea polyacrylamide gel showing the three TSSs (TSS1 TSS2 and TSS3). Lanes T, A, C and G show the sequencing ladder of respective bases and the lane PE shows primer extension products. The TATA-box is marked with a shaded elliptical shape and the bands corresponding to the TSS are represented by elliptical borders. A portion of the DNA sequence downstream of TATA-Box is written besides the autoradiogram (as derived from the sequencing ladder) and the TSS bases are represented with bend arrows (showing the direction of the transcription). The distance of TSS1 (taken as +1) to the TATA-Box is also shown. (C) Schematic representation of the TATA-box and the observed TSS in the genomic DNA upstream of ATG codon of *rpL32_8.1* gene. The underlined sequence represents the intron in the 5′UTR portion of the gene. The shaded arrow over the sequence represents the primer used in the primer extension. ATG start codon is boldly marked.

To further confirm the transcriptional downregulation of *rpL32_8.1,* promoter:reporter assays were performed. A ∼1.23 kb (approx), DNA fragment corresponding to the promoter region of *rpL32_8.1* gene was PCR amplified using genomic DNA from Pokkali indica rice variety and cloned in TA cloning vector. The sequence of this fragment did not show any significant difference from the sequence of the same region in japonica rice, which is available at RGAP 6.1 database (Fig. 5A). An *in silico* search using PLACE (http://www.dna.affrc.go.jp/PLACE/) indicated the presence of various *cis-*elements within this region (Fig. S5). To determine the transcription start site (TSS) in this cloned fragment, a primer extension assay was carried out using total RNA from shoots. The result indicated the presence of three major TSSs which were named as TSS1, TSS2 and TSS3 (Fig. 5B,C). The TSS1 was considered as the +1 TSS. No conventional TATA-box was found in the vicinity of the TSSs, but a TATA-like sequence TATAAGA was found 27 bp upstream of the +1 TSS (Fig. 5B,C).

**Figure 6:**
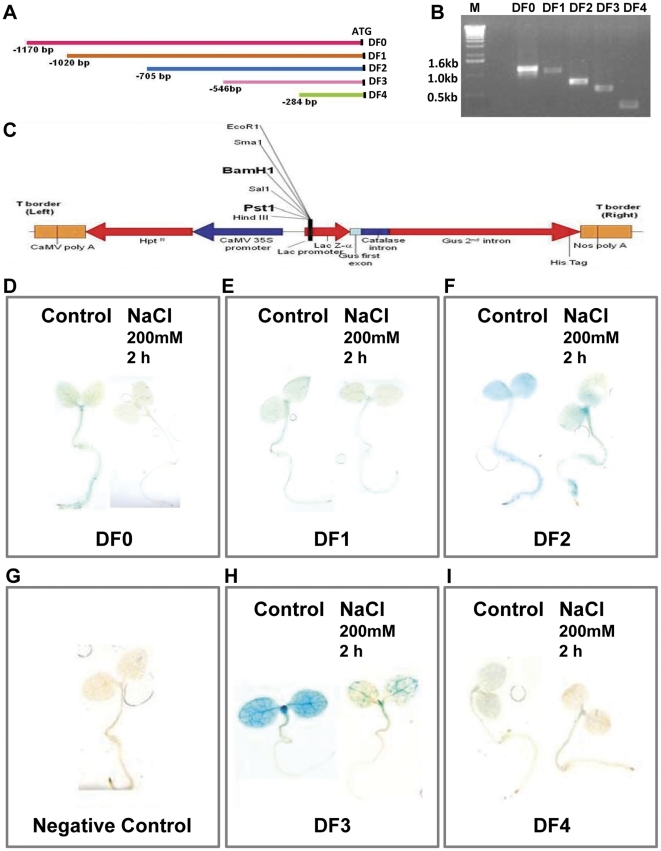
Analysis of *rpL32_8.1* promoter for salt responsiveness. (A) Schematic representation depicting the length of different promoter deletion fragments upstream of ATG start codon. (B) Agarose gel image showing PCR amplification of the different promoter fragments. (C) T-DNA portion of pCAMBIA1391z. *Bam*HI and *Pst*I sites were used for cloning of different promoter fragments. (D) – (I) Photographs of T1 generation transgenic seedlings showing GUS histochemical staining with X-Gluc. The different conditions and construct names are mentioned in each photograph. The wild type tobacco plant was used as negative control.

To identify the minimum functional promoter region responsible for the expression and stress responsiveness of *rpL32_8.1*, promoter:GUS constructs were prepared in pCAMBIA 1391z vector (Fig. 6A–C) using a 1170 bp DNA fragment (DF0; considered as the full length promoter fragment) and its four serial deletions (DF1, DF2, DF3 and DF4). These promoter:GUS constructs were then used to raise transgenics of tobacco. Seeds collected from the GUS-positive tobacco plants were germinated on medium containing hygromycin and GUS histochemical staining was done after 2 h of transferring one-week-old seedlings to medium containing 200 mM NaCl or lacking it. The assay was performed for 3–5 different transgenic lines of each of the promoter fragments and the results are summarized with a representative picture for each transgenic (Fig. 6D–I). Plants bearing DF0 and DF1 fragments hardly showed any GUS staining in the presence or absence of salt stress (Fig. 6D,E). However, DF2 transgenic plants showed a strong GUS expression in both shoots and roots which decreased significantly in shoots upon salt treatment (Fig. 6F). DF3 transgenics also showed a strong expression of GUS but only in shoots which also decreased upon stress treatment (Fig. 6H). Plants bearing DF4 fragment showed a faint GUS expression in shoots which further decreased when subjected to salt stress (Fig. 6I). Thus, the promoter:GUS assays indicate that the transcriptional regulation of *rpL32_8.1* gene originates within the promoter region of the gene. A significantly larger difference of GUS expression in unstressed shoots of DF3 and DF4 plants indicated that *cis-*elements required for the higher level of transcription of this gene are concentrated within a 262 bp region in the promoter (region which is present in DF3 fragment but not in DF4). Though the DF4 fragment expressed GUS faintly, it downregulated the expression of the reporter when subjected to stress. It is possible that either a repressor element is present within DF4 fragment, or the downregulation of the associated gene is caused by removal of transcription factors from the *cis-*elements in DF3 and DF4 fragments. For further analysis, this 262 bp DNA region was studied to determine if it was indeed the binding region of transcription factors.

### Downregulation of *rpL32_8.1* gene is associated with removal of transcription factors

**Figure 7:**
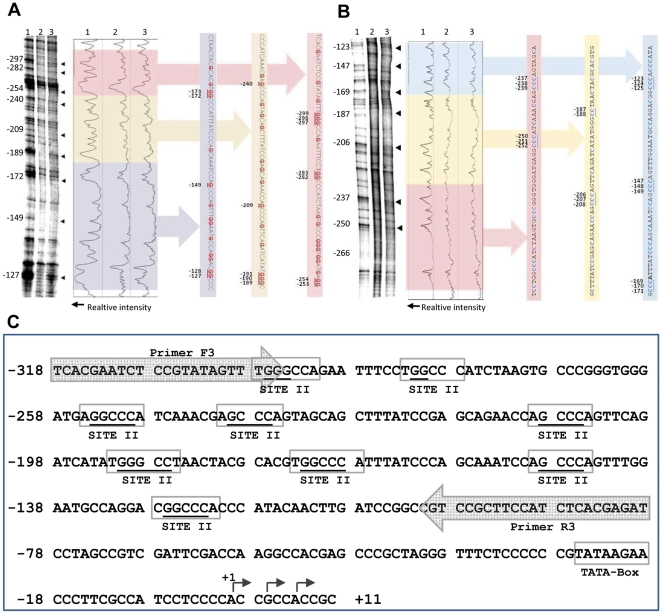
DMS-LMPCR in vivo footprinting of the promoter region of *rpL32_8.1*. (A) *In vivo* footprinting of the top strand. Lane 1 shows the LMPCR products of *in vitro* DMS-treated piperidine fragmented genomic DNA (experimental control). Lanes 2 and 3 show the *in vivo* DMS-LMPCR generated products from unstressed and stressed (200 mM NaCl, 24 h) shoot samples, respectively. The distance of the respective bases from the +1 TSS is shown on the left side of the autoradiogram. The arrow heads on the right side of the autoradiogram show the sites of differential protein binding. An uncalibrated graph (drawn using ImageJ software) showing densitometric quantification of bands in each lanes is presented besides the autoradiogram. DNA sequence of the top strand corresponding to different regions of the autoradiogram has also been depicted. The underlined ‘G’ residues are the sites showing differences in the footprinting pattern and correspond to the arrow heads. (B) *In vivo* footprinting of the bottom strand. All representations are same as mentioned for (A)., ‘C’ residue in the top strand are marked in place of ‘G’ residues as the footprinting was done for the bottom strand. (C) Schematic representation of the *cis-*elements showing differential binding of transcription factors under control and stressed conditions (indicated by underlined ‘G’ and ‘C’ residues). The possible *cis-*elements have been represented by shaded boxes with their names. The location of TATA-Box, TSS and the third primer (R3 and F3) used in LMPCR are also shown. The number at the start of each sequence line is the base pair distance from the first TSS (+1 site).

DMS-LMPCR *in vivo* footprinting was used to investigate if any *cis-*element in the aforementioned 262 bp region was showing differential *trans*-factor binding in the presence and absence of salt. A marked difference in protein binding was observed between stressed (200 mM NaCl, 24 h.) and unstressed (control) samples, at many ‘G’ residues in the top and bottom strand of the scanned region of the promoter fragment (Fig. 7A, B). In all these sites, a factor binding under the control condition (Lane 2; Fig. 7A,B) was present in reduced amounts under salt stress (Lane 3; Fig. 7A,B) which was evident from the appearance of bands in the latter case (pointed by arrowheads). LMPCR products of piperidine cleaved *in vitro* DMS-treated genomic DNA was used as experimental control in this assay (Lane 1; Fig. 7A,B). By superimposing ‘G’ residues detected by *in vivo* footprinting of top and bottom strands, over the sequence of the promoter region, three different core sequences showing differential binding of transcription factors between control and stressed samples were identified (Fig. 7C). These were 5′GGCCC3′ (at −282, −254, −173 and −127), 5′GCCC3′ (at −240, −209 and −149), and 5′GGGCC3′ (at −297 and −191). Differential binding of transcription factors on 5′GGGCC3′ element at −297 bp and 5′GGCCC3′ element at −282 bp was detected only in top strand footprinting.

It was observed that all the functional GGCCC sequences are followed by an ‘A’ residue (Fig. 7C). Similarly, all GGGCC sequences are preceded by a ‘T’ residue and GCCC sequences are flanked by an ‘A’ residue on each side. Hence, the functional *cis-*elements were considered as GGCCCA, TGGGCC, and AGCCCA. The sequences GGCCCA and TGGGCC are reverse complementary to each other and hence were considered as the same *cis-*element. Upon comparing the sequences of the remaining two *cis-*elements, we could assign them with a consensus sequence of (A/G)GCCCA. In PLACE database, this consensus sequence is denoted as SITE II element.

**Figure 8:**
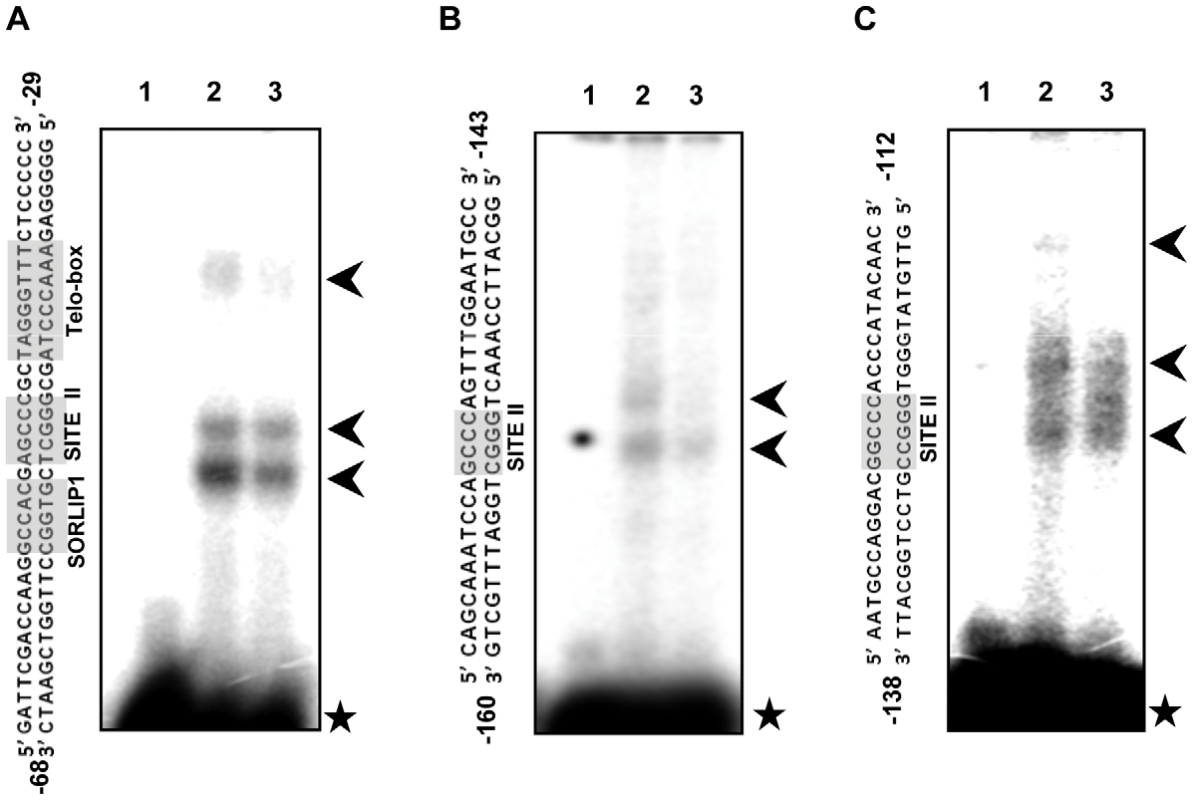
EMSA with nuclear extracts from untreated and salt stressed plants. (A) EMSA with a DNA oligo corresponding to a portion of DF4 fragment containing a SORLIP1 site, a SITE II like element and a telo-box. (B) EMSA with a DNA oligo containing AGCCCA element. (C) EMSA with a DNA oligo containing GGCCCA element. Lane 1 of (A), (B) and (C) represents the experimental control lane containing no protein. Lanes 2 and 3 include nuclear extracts from untreated and stressed (200 mM NaCl, 24 h) plants, respectively. The sequence of the double stranded oligos along with their relative position in the promoter with respect to the +1 TSS is mentioned beside each autoradiogram and the *cis-*elements are marked. The arrows indicate the gel shift bands and the star indicates the free probes.

The DF4 fragment, which expressed GUS very faintly and contributed to further downregulation of GUS under salt stress, was found to have a 40 bp DNA region containing a telo-box, a GCCCR type SITE II element and a SORLIP1 site. On performing an EMSA using this 40 bp DNA fragment and nuclear proteins from shoots of unstressed and stressed (200 mM NaCl, 24 h) rice seedlings, a reduction in protein binding was observed under stressed condition (Fig. 8A). Similar results were also obtained when two other oligos containing different SITE II elements, on which reduced protein binding was observed under salt stress by *in vivo* footprinting assays, were used in EMSA experiments (Fig. 8B,C). This substantiates the previous observations and indicates that the reduction in expression of *rpL32_8.1* is associated with removal of transcription factors from its promoter.

## Discussion

The mechanism of downregulation of genes encoding ribosomal proteins under stress conditions has not been well studied. The present work indicates that the genes encoding rpL32 are transcriptionally downregulated under abiotic stress in rice and this might be mediated via removal of transcription factors from the promoter region.

*In silico* analysis indicated that rpL32 is encoded by four genes in rice and belongs to a small multigene family as also reported earlier in plants and yeast [27]–[31]. The northern blots and RT-PCR analyses indicated that all *rpL32* genes are downregulated under stress conditions in shoots of rice seedlings. Changes in expression pattern of various ribosomal proteins have been reported from many plant systems under different stresses [6]–[8], [10]. In differential cDNA libraries of barley also, this gene was found to downregulate under abiotic stress [26]. It appears that downregulation of genes encoding rpL32 under stress is a common phenomenon in monocot plants.

Ribosomal proteins in plants have been reported to be translationally downregulated under osmotic stress via pathways involving TOR kinase, RAPTOR, PDK1 and S6K [12]–[15]. Post-transcriptional regulation of ribosomal proteins S4, S6, S28, L3, C24 (L13), L16 and P2, has also been suggested in plants [7], [18]–[21]. Despite identifying *cis*-elements and transcription factors responsible for high expression of ribosomal protein genes in growing and dividing plant cells [16], [17], none of the reports describes transcriptional regulation of these genes under stress. In the current study, using nuclear run-on assay transcriptional downregulation of all four genes of rice encoding rpL32 in response to salt stress was inferred. Relatively weak detection of *rpL32_9.1* in unstressed shoots during expression and nuclear run-on analyses might be due to restrictive transcription of this copy in a few specific cell types and/or at a different developmental stage, if not due to lower hybridization efficiency of the probe or the primers. In plants, variation in expression pattern among genes encoding the same ribosomal protein were also observed in previous studies [28], [31]. Being the major transcript observed in our experimental conditions, the regulatory studies under salt treatment were performed for *rpL32_8.1* gene.

Promoter-dependent stress-responsive transcriptional downregulation of *rpL32_8.1* was confirmed by promoter:GUS assays in transgenic tobacco plants. The failure of DF0 and DF1 fragments to express GUS might have occurred due to the absence of an activator element in the isolated 1.2 kb promoter fragment which might have a role in suppressing the activity of a repressor element within the DF1 region. In fact, a Matrix Attachment Region was predicted 5 kb upstream of the ATG start codon of *rpL32_8.1* gene.

The results of promoter:GUS assays suggested that majority of the *cis*-elements responsible for stronger expression of *rpL32_8.1* in unstressed shoots were located within the DF3 fragment; more specifically, between 284 bp and 546 bp region upstream of the ATG codon. An *in vivo* footprinting assay of this region indicated the presence of many (G/A)GCCCA elements where reduced binding of transcription factors was observed under salt stress. This observation was also confirmed by EMSA. (G/A)GCCCA (reverse complementary of TGGGC(C/T) sequences are variants of SITE II elements and are the binding sites of Class-I TCP-domain proteins. (G/A)GCCCA and GCCCR SITE II elements are enriched in promoters of ribosomal protein genes and have been reported to be necessary for high expression of ribosomal protein and other genes in actively growing and dividing cells of *Arabidopsis* and rice [16], [17], [32]–[35]. EMSA also detected reduced binding of proteins from shoots of stressed plants over a 40 bp portion of DF4 fragment containing a telo-box (TAGGGTTT), GCCCR type SITE II element (AGCCCG) and SORLIP1 site (GCCAC). This might be responsible for the decrease in GUS expression in DF4 transgenics under salt stress. Our work, hence, strongly suggests that the transcriptional downregulation of *rpL32_8.1* might be linked to the removal of transcription factors from the TCP-domain protein binding sites and other related elements. *In silico* analysis had also predicted the presence of GGCCC and GCCAC elements in promoters of stress downregulated genes [36]. It is being hypothesized that a similar mechanism might be mediating the decrease in transcription of other genes encoding rpL32 in rice under stress as similar *cis*-elements were also detected in their promoters by *in silico* methods. (Fig. S6).

Since many ribosomal proteins are coordinately regulated, a comparison was performed between the the abundance of (A/G)GCCCA elements within 1 kb region upstream of the ATG codon of different cytoplasmic ribosomal protein genes of rice and the level of expression of these genes under drought and salt stress. The expression data was obtained from Microarray Experiment ID GSE6901 (www.ricearray.org). Of 140 genes encoding ribosomal proteins, having a unique Affymetrix probe ID and at least two (A/G)GCCCA elements, 64.25% and 72.85% genes were found to downregulate (more than 1.2 times) under drought and salt stress, respectively. This indicates that (A/G)GCCCA elements are probably enriched in stress-downregulated genes (Fig. S7). However, the influence of other associated *cis-*elements on the promoter cannot be ignored as nearly 25–30% genes do not show significant change in expression under stress conditions (more than 1.2-fold up- or downregulation) and 4% of these genes were found to be upregulated significantly, even though they were highly enriched in (A/G)GCCCA elements (5 or more).

Expression analysis indicated relatively moderate decrease in *rpL32_8.1* transcripts in response to external sucrose treatment. No differential protein binding by *in vivo* footprinting was observed at the sugar-responsive elements (SRE) within the analyzed promoter fragment. In response to ABA no consistent decrease in the transcript level of *rpL32_8.1* was observed. These results indicated that sugar and ABA might not be directly involved in the stable downregulation of *rpL32_8.1* in rice under stress conditions.

Based on the available literature and the present study, it appears that ribosomal proteins are downregulated both at transcriptional and translational level under abiotic stress conditions. The rate of downregulation of *rpL32_8.1* transcripts seems to correlate with the severity of stress and degree of tolerance of the plant as the rate of downregulation was highest for drought stress and was higher in salt sensitive variety PB1. Gene(s) encoding rpL32 is present in all organisms ranging from archaea to higher eukaryotes and the sequence also shows high conservation among various groups of organisms (Fig. S8,S9), thereby indicating that this protein is needed for proper growth and development of all organisms. In fact in *Drosophila*, the deletion of this gene was found to result in various growth and developmental defects [22]. This could be the reason for its downregulation only transiently for the time for which the stress was applied and rapid return to its normal level as soon as the stress was withdrawn. *rpL32_8.1* was found to get downregulated in shoots but not in roots upon exposure to salinity stress. Though the exact mechanism is not known, differential expression of genes in different tissues under various abiotic stresses is well known. The stress perception and the signaling mechanisms do vary in different tissues of plants. The prevailing environmental factors, under which plants are subjected to salinity stress, might also generate differential signals like calcium in shoots and roots [37]. Whether the rate of downregulation of such ribosomal proteins determines the stress tolerance level of a plant variety, needs further experimentation.

## Materials and Methods

### Plant material, growth conditions and sample collection

Rice seedlings were grown under control conditions in growth chamber at 28±2°C and 16 h photoperiod in a hydroponics culture system supplied with modified Yoshida medium [38]. At three leaf stage, different treatments (dissolved in Yoshida medium) were given and tissues were collected at various time points. All experiments were done with indica rice variety Pokkali unless stated otherwise.

### Amplification and cloning of different genomic fragments

The 3′ UTR region of different *rpL32* genes and the promoter fragment of *rpL32_8.1* gene were amplified from the genomic DNA by two rounds of PCR (30 cycles each), the second one being done with a set of nested primers (see Table S1 and S2 for primer sequences) and cloned in TA-cloning vector (TOPO-TA, Invitrogen). The 5′ serial deletions of the promoter fragment were generated by PCR using a fixed reverse primer R1 and different forward primers (primers sequences are given in Table S3). These PCR products were cloned in pCAMBIA 1391z plant transformation vector between the *Pst*I and *Bam*HI sites to make the promoter:GUS constructs.

### Northern blotting

20 µg of total RNA (isolated by using Trizol reagent from Invitrogen) was blotted onto nylon membranes according to Sambrook et al [39]. The membrane was pre-hybridized for 6 h at 55–60°C in buffer containing 5X SSC, 5X Denhardt reagent, 0.1% SDS, 100 µg/ml denatured salmon sperm DNA and 10% dextran sulphate. Thereafter, radio-labeled pre-denatured probe (generated by PCR) was added to the pre-hybridization solution. After 16–18 h of incubation at 60°C in the hybridization solution, membranes were washed once with 2X SSC at room temperature, once with 2X SSC, 0.5% SDS for 15 min and twice with 0.5X SSC, 0.5% SDS for 15 min at the hybridization temperature. The blots were imaged using a phosphorimager (Typhoon Scanner; GE Lifescience). All densitometric analyses were done using ImageJ and Microsoft excel software.

### Semiquantitative RT-PCR

cDNA was prepared from 4 µg of total RNA using oligo dT cDNA synthesis kit (Fermentas). PCR conditions and required number of PCR cycles were standardized for each gene using 5-fold, 10-fold, 50-fold and 200-fold diluted cDNA. *rpL32_8.1* was amplified using 50-fold diluted cDNA for 26 cycles. *rpL32_9.2* and *rpL32_9.3* were amplified using 10-fold diluted cDNA for 27 cycles. *rpL32_9.1* was amplified using 5-folds diluted cDNA for 30 cycles. *EF-1α* was used as a control gene and was amplified using 10-fold diluted cDNA for 25 cycles. All PCRs were performed with 1 µl of the respective cDNA in 20 µl of reaction mixture. 10 µl of the amplified products were electrophoresed and imaged using gel doc. All further densitometric analyses were done using ImageJ and Microsoft excel software. The details of the primers used in RT-PCR analysis are mentioned in Table S4.

### Isolation of nuclei from rice shoots

Nuclei were isolated by minor modification of the protocols published earlier [40], [41]. Briefly, 10 g of frozen rice shoots were crushed to fine powder in liquid N_2_ and was homogenized with 100 ml of nuclei isolation buffer (10 mM PIPES, pH 7.0; 10 mM MgCl_2;_ 1 M hexylene glycol; 10 mM β-mercaptoethanol; 2.5% Ficoll; 0.5% triton X-100; 2 mM spermine). After filtration through 3 layers of cheese cloth and 2 layers of miracloth (Calbiochem), and centrifugation (3300 g for 8 min), the pellet was washed thrice with washing buffer (10 mM PIPES, pH 7.0; 10 mM MgCl_2;_ 1 M hexylene glycol; 10 mM β-mercaptoethanol), twice in presence and once in absence of triton X-100. Finally the nuclear pellet was resuspended in 500 µl of resuspension buffer (10 mM PIPES, pH 7.0; 10 mM MgCl_2;_ 1 M hexylene glycol; 10 mM β-mercaptoethanol; 25% glycerol), snap frozen in liquid N_2_ and stored at −80°C until used. DNA from the isolated nuclei were measured according to Wanner and Gruissem [42].

### Nuclear run-on assay

The nuclear run-on assay was done according to Hirose and Yamaya [43] and Kanazawa et al [44] using Rnasin-treated (with 100 units; Fermentas) 50 µg DNA equivalent nuclei preparation in 200 µl of 1X nuclear transcription buffer that includes 10 µl each of 10 mM ATP, GTP and CTP (Ambion) and 10 µl of α^32^P-UTP (3000Ci/m mol). The synthesized radiolabeled RNA was isolated in presence of 25 µg yeast t-RNA (Ambion) and equal counts of the heat denatured RNA were added to dot blot that were preincubated for 6 h at 55°C with 2 ml of hybridization buffer (250 mM sodium phosphate pH 7.2 and 100 µg/ml ssDNA). For preparing these dot blots, equal amount (500 ng) of PCR generated 3′ UTR portions of different *rpL32* genes and 18S rRNA, and pBSK plasmid was used. The blots were washed at 55°C in a similar way as was done for Northern hybridization and then imaged using a phosphorimager. All densitometric analyses were done using ImageJ and Microsoft excel software.

### Transcription start site (TSS) mapping

For mapping TSS, primer extension was done with cDNA prepared from 5-10 µg of total RNA using MMLV-Reverse Transcriptase H- (Fermentas) as per manufacturer's instruction and 50 ng of γ^32^P end labeled primer (5′-TTCGGATCGGAGAGGGAAGC-3′), designed from the 5′UTR just upstream of the first intron of *rpL32_8.1* gene. The primer annealing step was done at 55°C. The primer extension products were run in parallel to a marker generated by manual sequencing (Sanger's dideoxy method manual sequencing kit; USB) of the cloned promoter fragment of *rpL32_8.1* using the same primer (but unlabeled), on a 40 cm long 6% denaturing urea-PAGE gel at 60W power (∼2000 V) and were compared with each other to find the TSS.

### Tobacco transformation via *Agrobacterium*

Leaf discs (∼1 cm^2^) obtained from four-week-old tobacco plants (grown axenically) were infected with secondary culture of *Agrobacterium tumefaciens* (A_600_ 0.6–1.0) bearing recombinant constructs. Shoot regeneration was done in 1X MS-Agar (including vitamins) containing 0.1 mg/L NAA and 1 mg/L BAP 250 mg/L carbenicillin, 25 mg/L hygromycin. Rooting was obtained in 1X MS-Agar (without vitamin and hormones). GUS histochemical staining and PCR with *uidA* specific primers was done from leaves to confirm transgenics. The seeds, collected from these T_0_ plants, were germinated on filter sheets soaked with 1X MS medium containing 50 mg/L hygromycin. One-week-old seedlings were treated with 200 mM NaCl solution for 2 h and GUS histochemical staining was carried out for salt treated and untreated seedlings.

### GUS histochemical staining assay

Seedlings were first washed twice with 50 mM sodium phosphate pH 7.0. The GUS staining buffer (50 mM sodium phosphate pH 7.0, 0.5 mM potassium ferrocyanide, 0.5 mM potassium ferricyanide, 0.1% triton X-100 and 1 mg/ml X-Gluc; Bioworld) was then vacuum infiltrated (for 10–15 min) into the samples. After this the samples were incubated in the same buffer under dark conditions at 37°C till the blue color was developed. The chlorophyll from the samples was bleached away using 70% ethanol before being photographed.

### In vivo Dimethyl sulfate-Ligation mediated PCR (DMS-LMPCR) footprinting

The DMS *in vivo* footprinting was done according to Busk and Pages [45]. Sequence of the linker primers (Linker1 and Linker2) and the primers for the blunt end generation, amplification and the final extension of top strand (R1, R2 and R3, respectively) and bottom strand (F1, F2 and F3) LMPCR are given in Table S5. For all steps of the LMPCR, Vent polymerase was used with 4 mM MgSO_4_ in the LMPCR buffer (20 mM Tris-HCl (pH 8.9), 40 mM NaCl, 0.009% NaCl and 0.09% Triton X-100). The amplification step was done for 18 cycles. For the final extension step, γ^32^P-dATP end labeled primer F3 and R3 were used. After electrophoresing, the gel was dried and imaged using a phosphorimager. Sanger's dideoxy sequencing reaction products of the cloned promoter fragment using the final extension primer was used as markers. The position of n^th^ guanidine residue in the footprinting lane was determined as a measure of its distance from the +1 TSS by deducting 25 nucleotides from the position of coinciding base in the marker lane because the LMPCR products only contained the 25 bp linker DNA.

### Electrophoretic mobility shift assay (EMSA)

10 µg of nuclear proteins, prepared according to He et al [46] were incubated with γ-^32^P end labeled double stranded oligos (50000 cpm) for 30 min in presence of 10 mM HEPES (pH 7.5), 1 mM MgCl_2_, 50 mM KCl, 10% glycerol, 0.5 mM DTT, 0.5 mM PMSF and 1 µg poly dI-dC in a 20 µl binding reaction at room temperature. The products were then run on a non-denaturing 4.5% polyacrylamide gel and imaged using a phosphorimager.

## Supporting Information

Figure S1 **Rice genome contains four genes encoding rpL32.** One of the genes is located on chromosomes 8 (*rpL32_8.1*) and three others on chromosome 9 (*rpL32_9.1*, *rpL32_9.2* and *rpL32_9.3*). The accession numbers as mentioned in the RGAP 6.1 database for each of these genes is shown in the figure. ORF of *rpL32_9.2* and *rpL32_9.3* are present on minus strand and are reverse in orientation.
(TIF)

Figure S2 ***rpL32*** **genes are similar in their CDS, predicted protein sequence and gene structure.** (A) ClustalW alignment of the CDS of the four *rpL32* genes. The top sequence represents *rpL32_8.1*, followed by *rpL32_9.1*, *rpL32_9.2* and then *rpL32_9.3,* respectively. (B) ClustalW alignment of the predicted protein sequence of the same. (C) Gene structure of the four *rpL32* genes as represented in the RGAP 6.1 database. *rpL32_9.2* and *rpL32_9.3* are present in reverse orientation in the genome. Their orientation has been changed to make the comparison. For *rpL32_9.1* and *rpL32_9.2*, two alternative spliced forms involving the first intron is predicted. The blue box represents exons.
(TIF)

Figure S3 **ClustalW alignment of the 3′UTR region of the four** ***rpL32*** **genes of rice.**
(TIF)

Figure S4 **Semiquantitative RT-PCR analysis of** ***rpL32*** **genes under different conditions.** (A) Analysis of *rpL32_8.1*, *rpL32_9.1*, *rpL32_9.2* and *rpL32_9.3* expression under salt stress (200 mM NaCl, 24 h) in shoots of PB1 rice variety. (B) Expression analysis of *rpL32_8.1* under salt stress in shoots of Pokkali rice variety. (C) RT-PCR analysis of *rpL32_8.1* under salt stress in roots of PB1 rice variety. (D) Expression analysis of *rpL32_8.1* in shoots of Pokkali rice during stress recovery. (E) RT-PCR analysis of *rpL32_8.1* under drought stress (air dried) and ABA(100 µM) treatment in shoots of pokkali rice. (F) Expression analysis of *rpL32_8.1* under cold stress (4°C) and sucrose (4.5%) treatment in shoots of pokkali rice. t0 to t24 indicates the time of treatment (in h). t24-1 to t24-24 indicates the time of incubation (in h) of plants under control condition after being subjected to salt stress for 24 h. The densitometric quantification values of *rpL32* genes were normalized with that for *EF1-α.* All analyses were performed with three technical and three biological replicates. The error bars in the histograms represent the standard deviation. Representative gel images of two biological replicates are shown in each case.
(TIF)

Figure S5 **Important** ***cis*** **-elements presents in the putative promoter region of** ***rpL32_8.1*** **.** The sequence belongs to the top strand and is presented in the 5′ to 3′ direction. The A residue at position 1020 represents (written in bold) the expected ATG start codon of the gene. The first intron and the 5′UTR region is also shown. Different *cis*-elements have been provided different colors with their names mentioned below their sequence.
(TIF)

Figure S6 **SITE II and SORLIP1 elements in the promoters of** ***rpL32_9.1*** **,** ***rpL32_9.2*** **and** ***rpL32_9.3.***
(TIF)

Figure S7 **Stress induced expression change and the abundance of (A/G)GCCCA elements in ribosomal protein genes.** Analysis presented here is of 140 genes encoding cytoplasmic ribosomal protein having unique Affymetrix probe ID and at least two (A/G)GCCCA elements. The top and middle graph shows the fold-change in expression of genes under drought and salt stress, respectively. The lower graph shows the abundance of (A/G)GCCCA elements in the promoter of these genes. The genes have been categorized on the basis of the number of (A/G)GCCCA elements in the promoter region as represented by vertical dashed lines and the corresponding abundance of the *cis*-element is also written below the lower graph. The red dashed lined box in the top two graphs represents insignificant change in gene expression (<1.2 fold up- or downregulation). A lower level of fold-change in fold expression was considered as significant as this microarray was done at a early time point and also includes roots where down regulation of *rpL32_8.1* was not observed.
(TIF)

Figure S8 **Guide tree showing the presence of rpL32 in different groups of higher eukaryotes.**
(TIF)

Figure S9 **A ClustalW alignment showing similarity between** ***rpL32*** **genes from different organisms. Tc**-*Tribolium castaneum*, **Dm**-*Drosophila melanogaster*, **Ag**-*Anopheles gambiae* str. PEST, **Hs**-*Homo sapiens*, **Mm**-*Mus musculus*, **Xt**-*Xenopus tropicalis*, **Os**-*Oryza sativa*, **Zm**-*Zea mays*, **At**-*Arabidopsis thaliana*, **Cr**-*Chlamydomonas reinhardtii*, **Sc**-*Saccharomyces cerevisiae*, **Sp**-*Schizosaccharomyces pombe*, **Ss**-*Sclerotinia sclerotiorum*, **Pat**-*Paramecium tetraurelia* strain d4-2, **Pfu**-*Pyrococcus furiosus* DSM 3638, **Tk**-*Thermococcus kodakarensis* KOD1, **Mk**-*Methanopyrus kandleri* AV19.
(TIF)

Table S1 **List of primers used for 3′ UTR cloning and amplification.**
(DOC)

Table S2 **List of primers used for promoter cloning of** ***rpL32_8.1*** **gene.**
(DOC)

Table S3 **List of primers used for generating promoter deletions of** ***rpL32_8.1*** **gene.**
(DOC)

Table S4 **List of primers used for semiquantitative RT-PCR analysis.**
(DOC)

Table S5 **List of primers used for** ***in vivo*** **DMS-LMCR footprinting.**
(DOC)
