# Supplementary figures and images for: Transcriptional Downregulation of Rice rpL32 Gene under Abiotic Stress Is Associated with Removal of Transcription Factors within the Promoter Region
Source: PLoS One. 2011 Nov 23;6(11):e28058. doi: 10.1371/journal.pone.0028058 (PMC3223225; doi:10.1371/journal.pone.0028058)

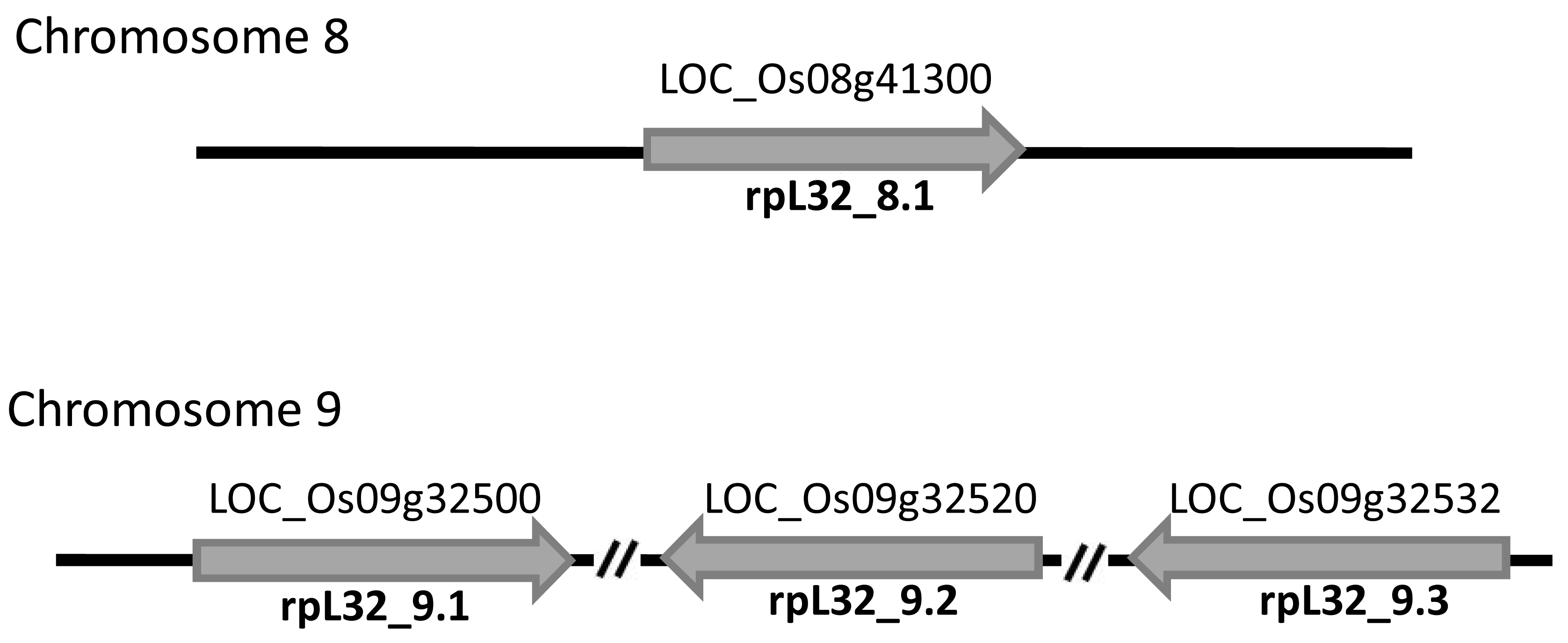

Supplement: Figure S1 — Rice genome contains four genes encoding rpL32. One of the genes is located on chromosomes 8 (rpL32_8.1) and three others on chromosome 9 (rpL32_9.1, rpL32_9.2 and rpL32_9.3). The accession numbers as mentioned in the RGAP 6.1 database for each of these genes is shown in the figure. ORF of rpL32_9.2 and rpL32_9.3 are present on minus strand and are reverse in orientation. (TIF) [file pone.0028058.s001.tif]

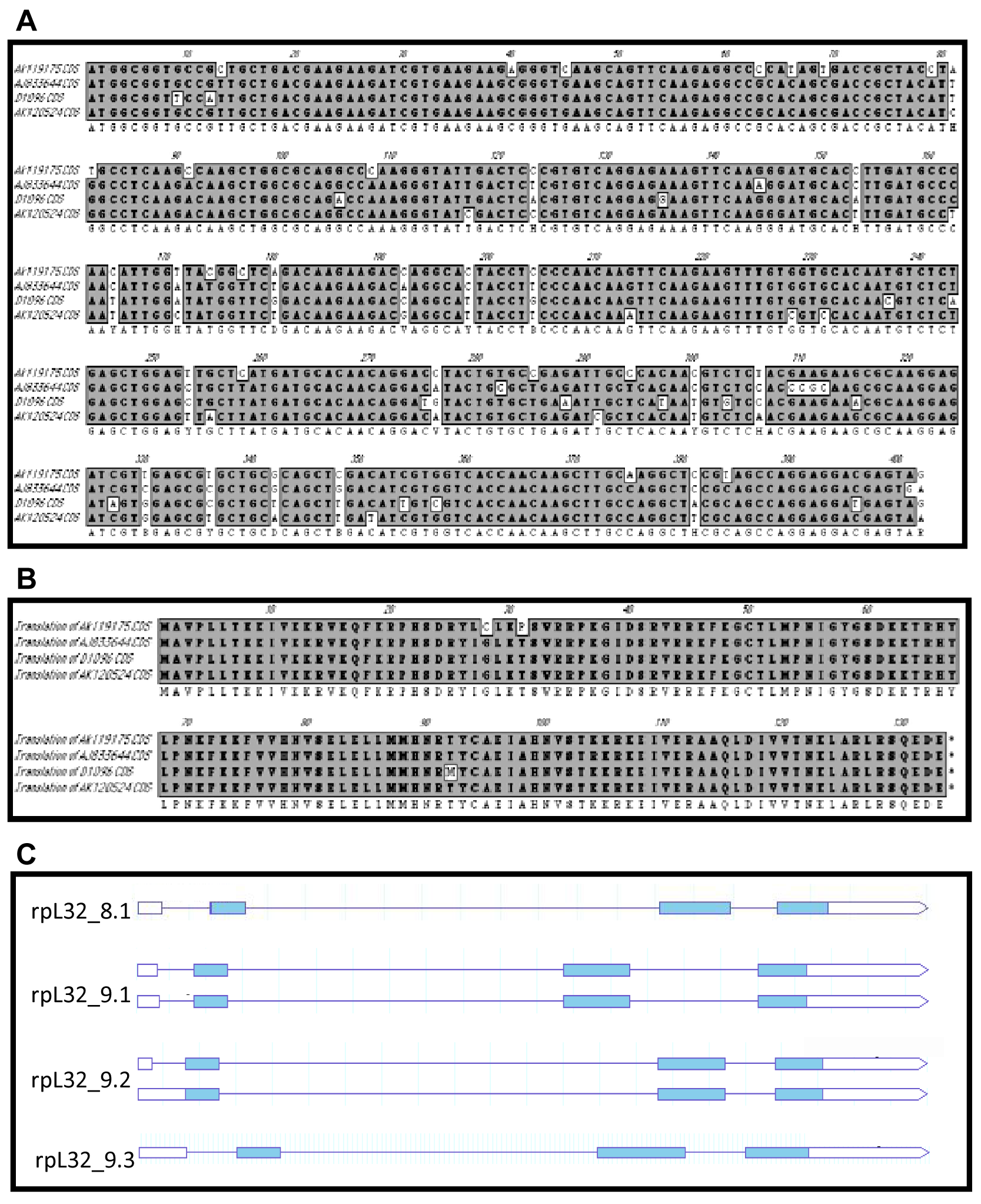

Supplement: Figure S2 — rpL32 genes are similar in their CDS, predicted protein sequence and gene structure. (A) ClustalW alignment of the CDS of the four rpL32 genes. The top sequence represents rpL32_8.1, followed by rpL32_9.1, rpL32_9.2 and then rpL32_9.3, respectively. (B) ClustalW alignment of the predicted protein sequence of the same. (C) Gene structure of the four rpL32 genes as represented in the RGAP 6.1 database. rpL32_9.2 and rpL32_9.3 are present in reverse orientation in the genome. Their orientation has been changed to make the comparison. For rpL32_9.1 and rpL32_9.2, two alternative spliced forms involving the first intron is predicted. The blue box represents exons. (TIF) [file pone.0028058.s002.tif]

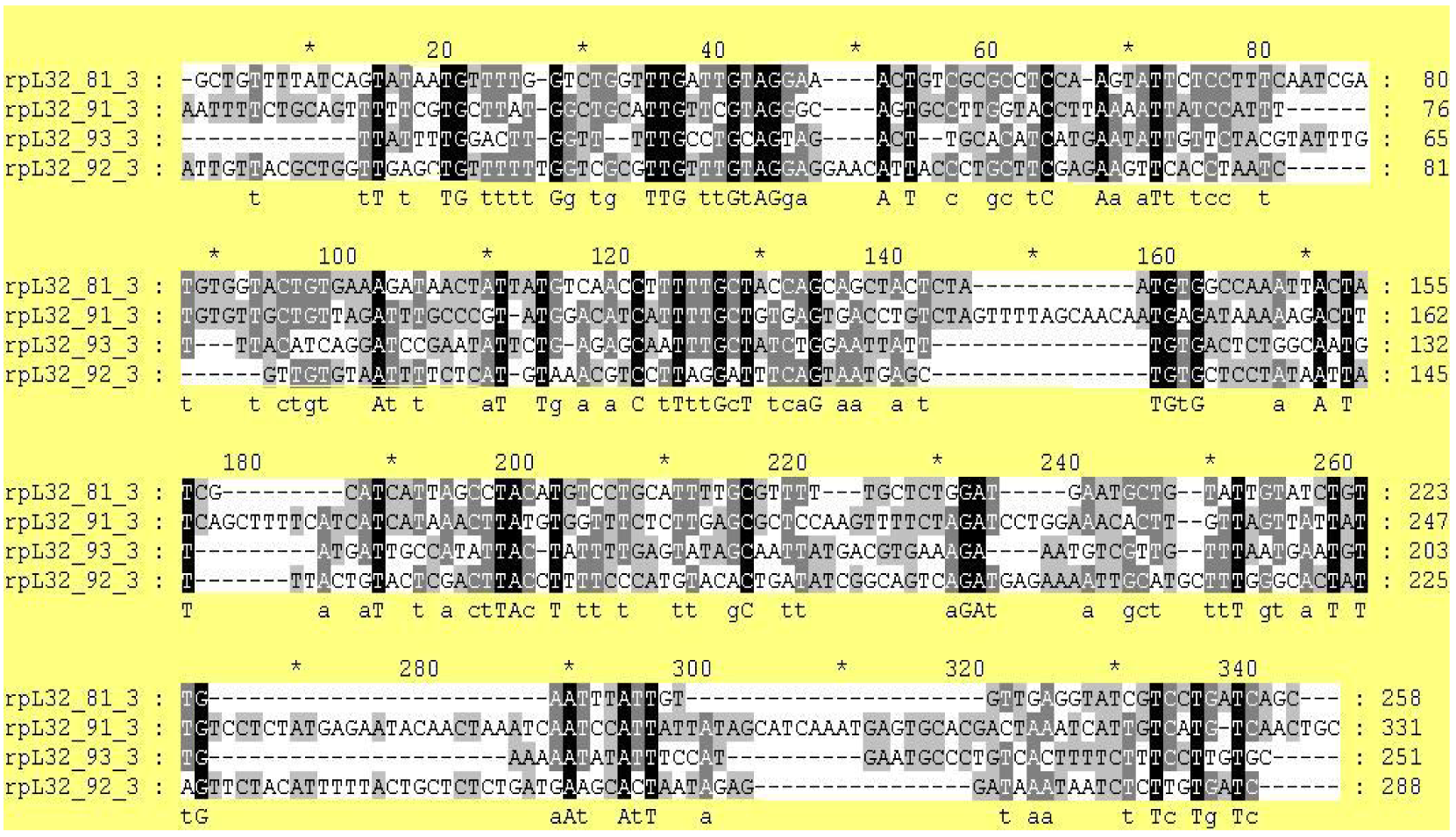

Supplement: Figure S3 — ClustalW alignment of the 3′UTR region of the four rpL32 genes of rice. (TIF) [file pone.0028058.s003.tif]

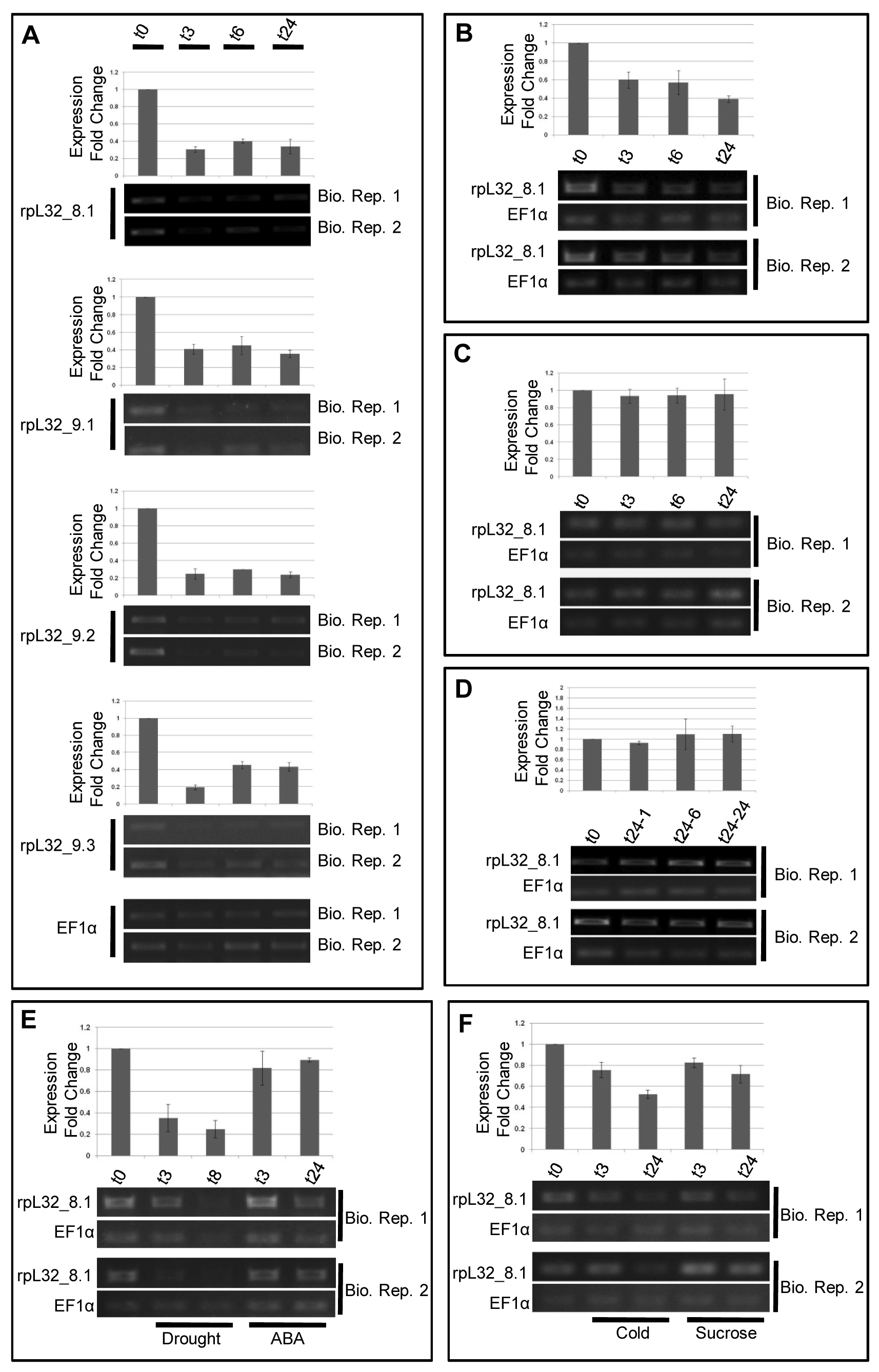

Supplement: Figure S4 — Semiquantitative RT-PCR analysis of rpL32 genes under different conditions. (A) Analysis of rpL32_8.1, rpL32_9.1, rpL32_9.2 and rpL32_9.3 expression under salt stress (200 mM NaCl, 24 h) in shoots of PB1 rice variety. (B) Expression analysis of rpL32_8.1 under salt stress in shoots of Pokkali rice variety. (C) RT-PCR analysis of rpL32_8.1 under salt stress in roots of PB1 rice variety. (D) Expression analysis of rpL32_8.1 in shoots of Pokkali rice during stress recovery. (E) RT-PCR analysis of rpL32_8.1 under drought stress (air dried) and ABA(100 µM) treatment in shoots of pokkali rice. (F) Expression analysis of rpL32_8.1 under cold stress (4°C) and sucrose (4.5%) treatment in shoots of pokkali rice. t0 to t24 indicates the time of treatment (in h). t24-1 to t24-24 indicates the time of incubation (in h) of plants under control condition after being subjected to salt stress for 24 h. The densitometric quantification values of rpL32 genes were normalized with that for EF1-α. All analyses were performed with three technical and three biological replicates. The error bars in the histograms represent the standard deviation. Representative gel images of two biological replicates are shown in each case. (TIF) [file pone.0028058.s004.tif]

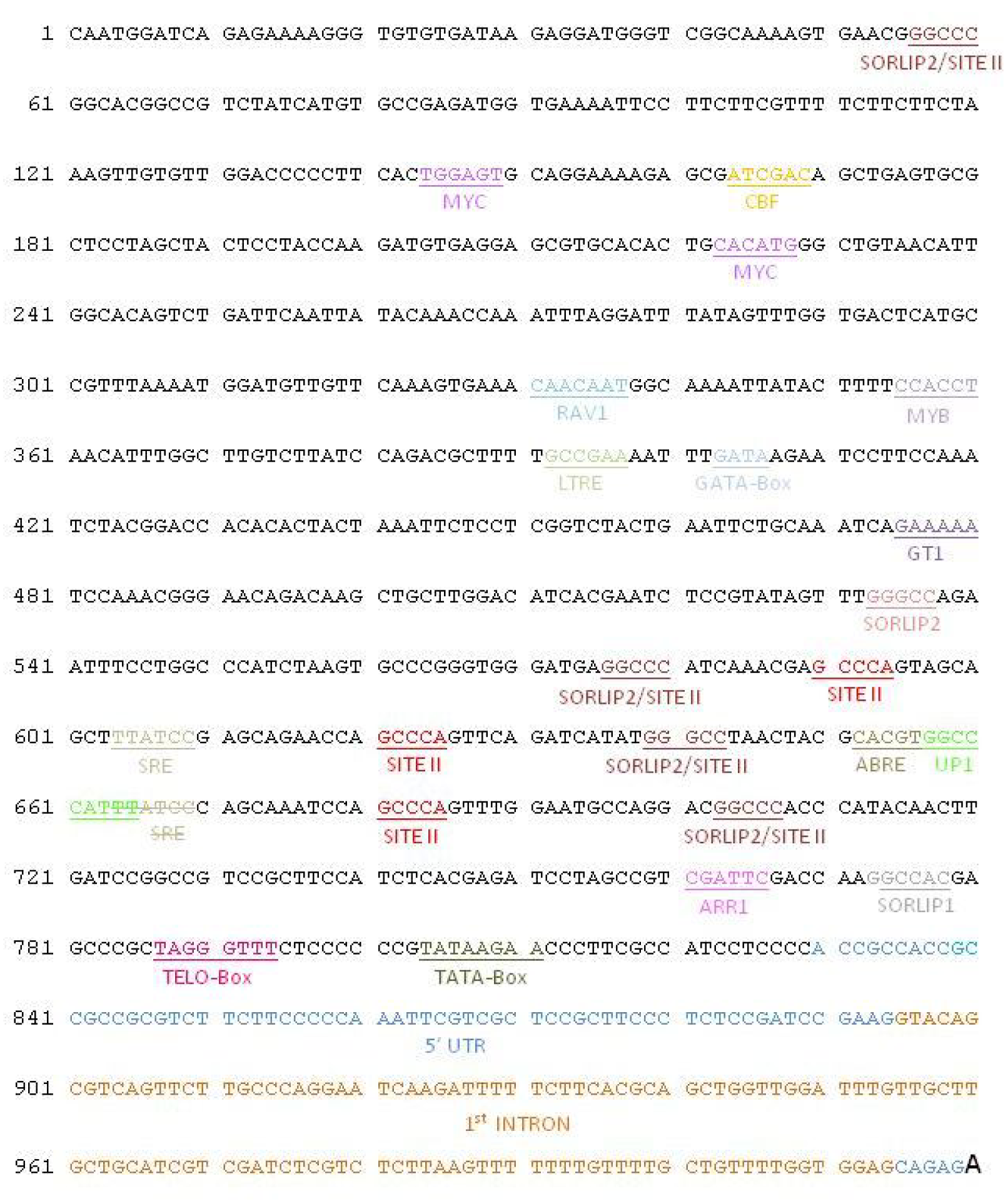

Supplement: Figure S5 — Important cis -elements presents in the putative promoter region of rpL32_8.1 . The sequence belongs to the top strand and is presented in the 5′ to 3′ direction. The A residue at position 1020 represents (written in bold) the expected ATG start codon of the gene. The first intron and the 5′UTR region is also shown. Different cis-elements have been provided different colors with their names mentioned below their sequence. (TIF) [file pone.0028058.s005.tif]

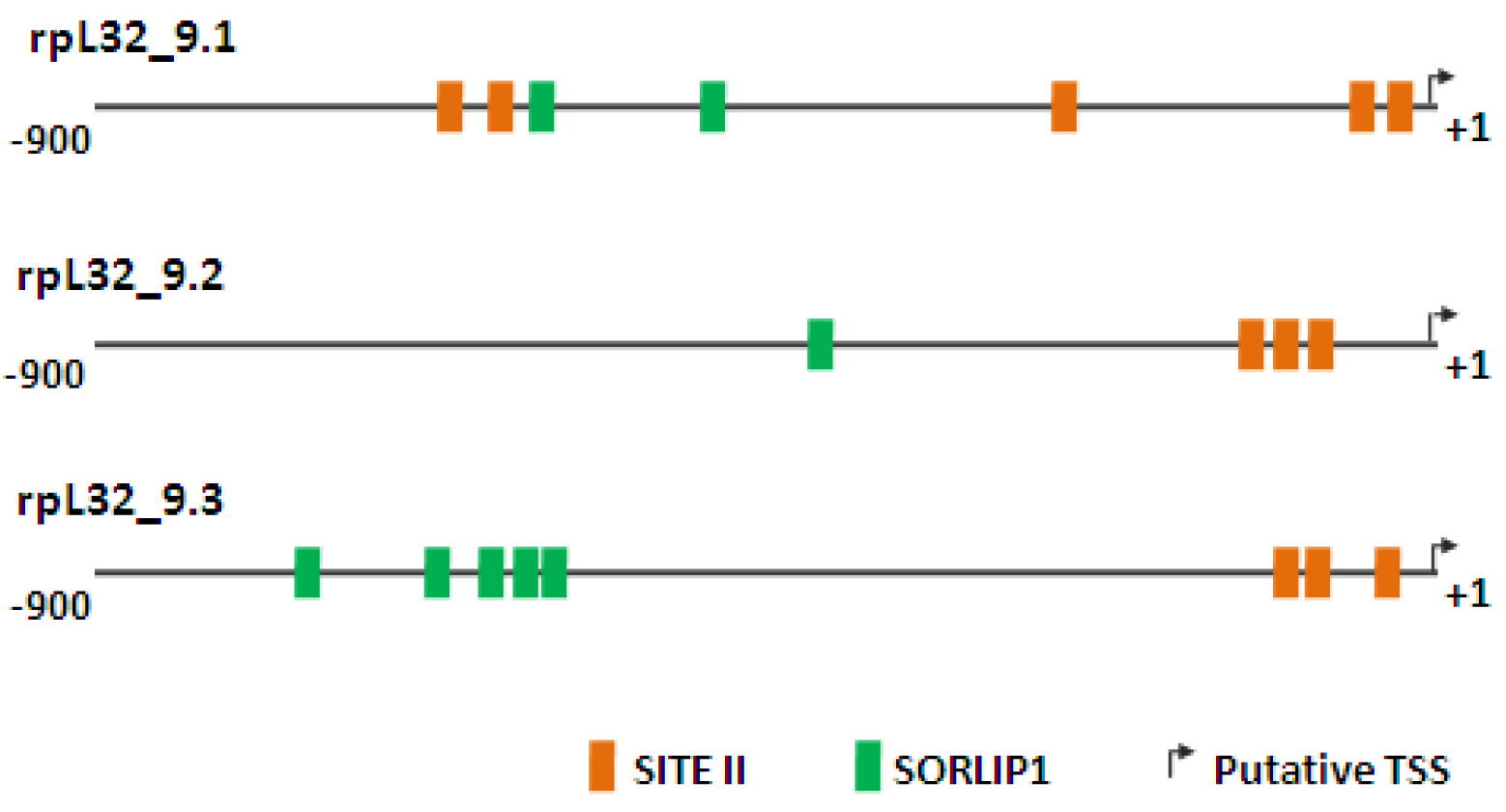

Supplement: Figure S6 — SITE II and SORLIP1 elements in the promoters of rpL32_9.1 , rpL32_9.2 and rpL32_9.3. (TIF) [file pone.0028058.s006.tif]

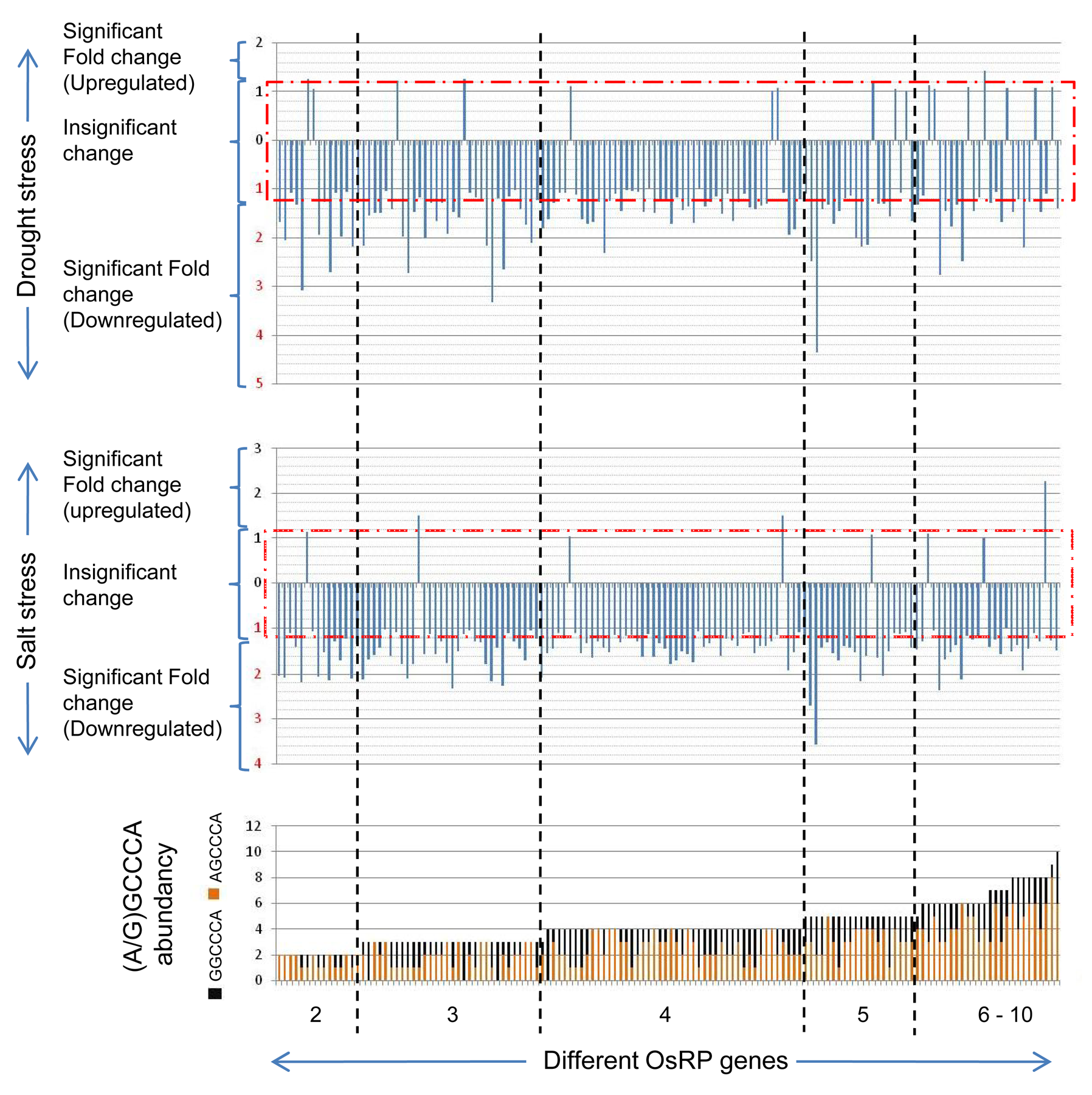

Supplement: Figure S7 — Stress induced expression change and the abundance of (A/G)GCCCA elements in ribosomal protein genes. Analysis presented here is of 140 genes encoding cytoplasmic ribosomal protein having unique Affymetrix probe ID and at least two (A/G)GCCCA elements. The top and middle graph shows the fold-change in expression of genes under drought and salt stress, respectively. The lower graph shows the abundance of (A/G)GCCCA elements in the promoter of these genes. The genes have been categorized on the basis of the number of (A/G)GCCCA elements in the promoter region as represented by vertical dashed lines and the corresponding abundance of the cis-element is also written below the lower graph. The red dashed lined box in the top two graphs represents insignificant change in gene expression (<1.2 fold up- or downregulation). A lower level of fold-change in fold expression was considered as significant as this microarray was done at a early time point and also includes roots where down regulation of rpL32_8.1 was not observed. (TIF) [file pone.0028058.s007.tif]

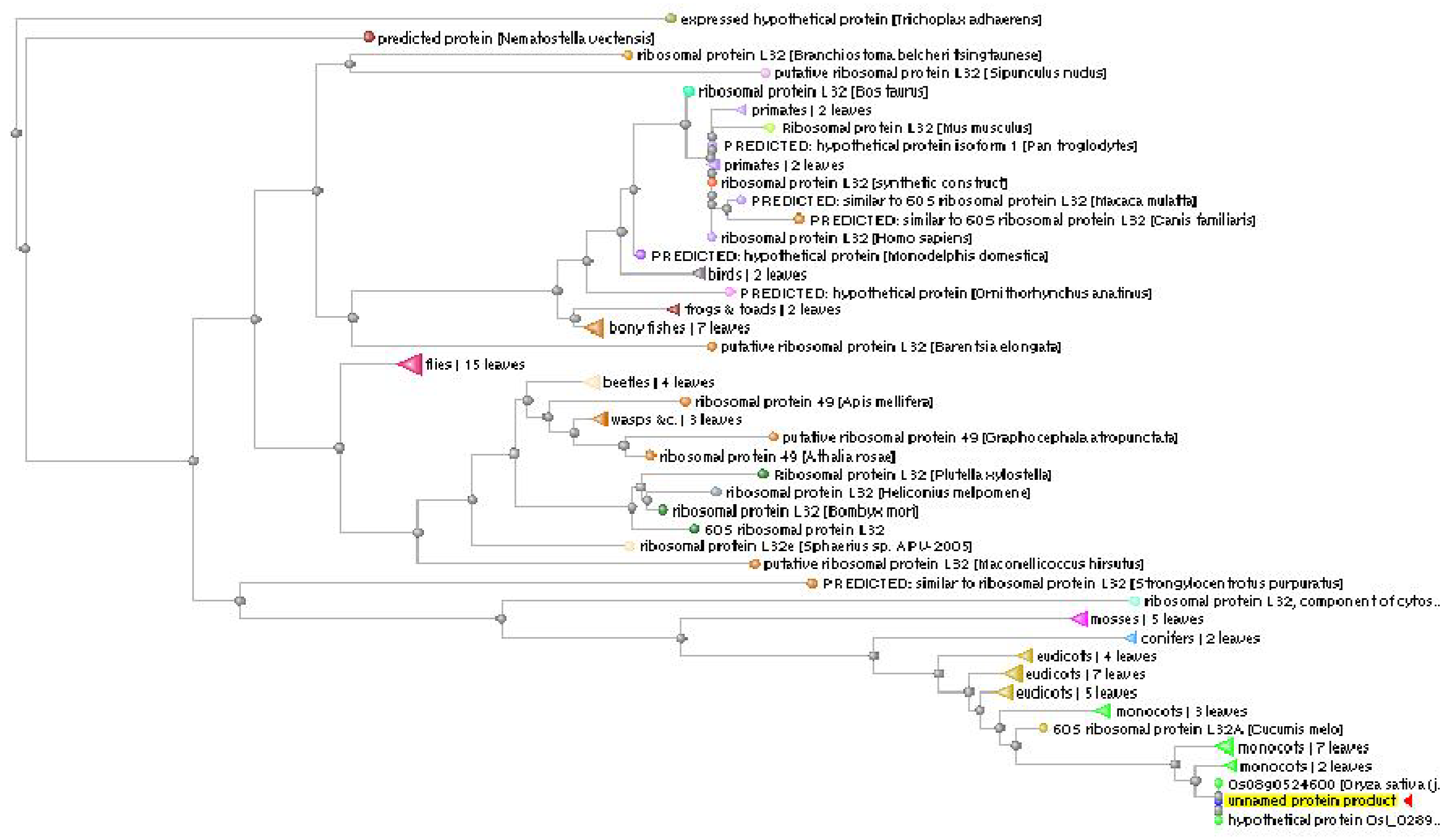

Supplement: Figure S8 — Guide tree showing the presence of rpL32 in different groups of higher eukaryotes. (TIF) [file pone.0028058.s008.tif]

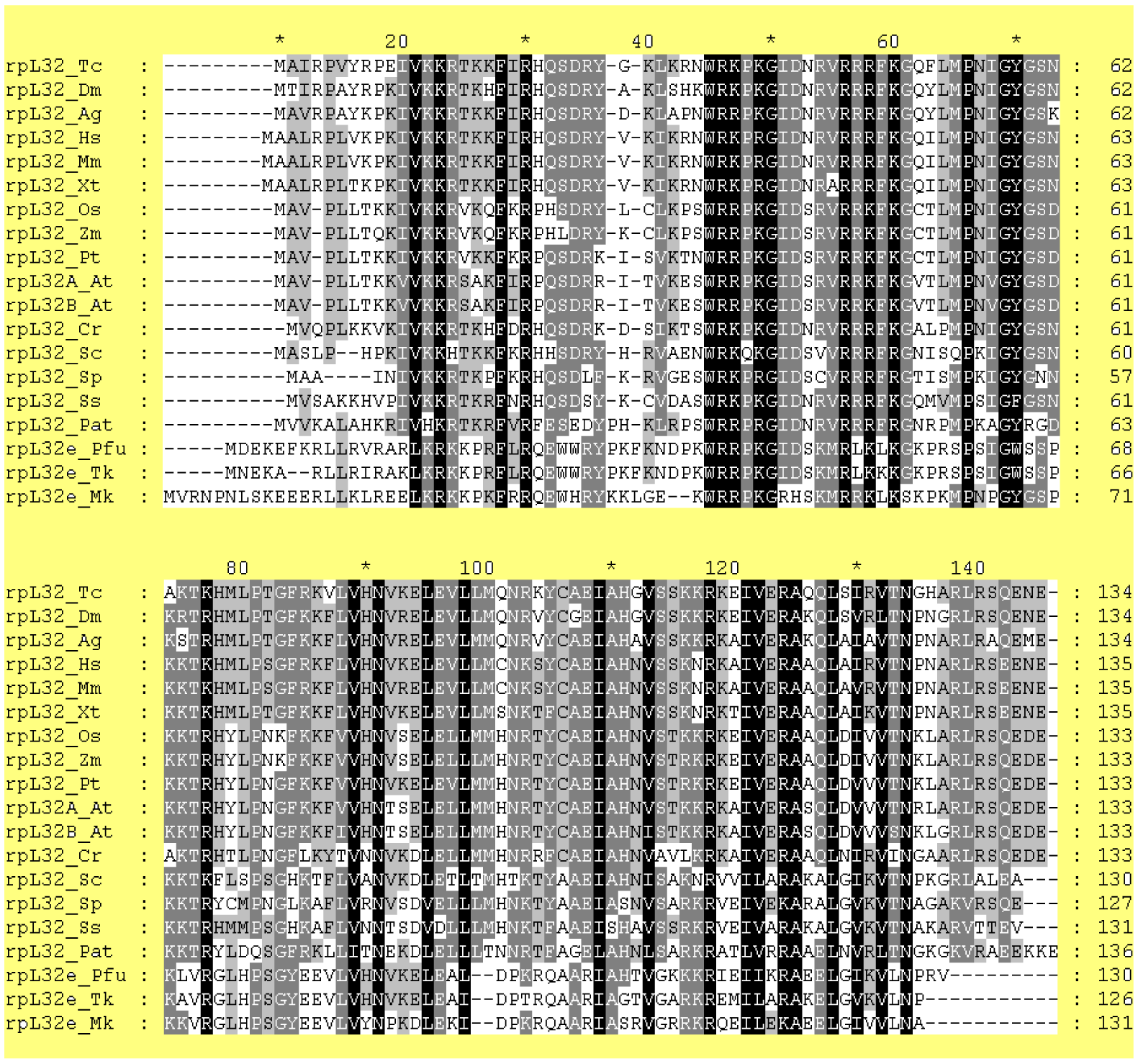

Supplement: Figure S9 — A ClustalW alignment showing similarity between rpL32 genes from different organisms. Tc-Tribolium castaneum, Dm-Drosophila melanogaster, Ag-Anopheles gambiae str. PEST, Hs-Homo sapiens, Mm-Mus musculus, Xt-Xenopus tropicalis, Os-Oryza sativa, Zm-Zea mays, At-Arabidopsis thaliana, Cr-Chlamydomonas reinhardtii, Sc-Saccharomyces cerevisiae, Sp-Schizosaccharomyces pombe, Ss-Sclerotinia sclerotiorum, Pat-Paramecium tetraurelia strain d4-2, Pfu-Pyrococcus furiosus DSM 3638, Tk-Thermococcus kodakarensis KOD1, Mk-Methanopyrus kandleri AV19. (TIF) [file pone.0028058.s009.tif]
