# Supplementary material for: Transcriptional Downregulation of Rice rpL32 Gene under Abiotic Stress Is Associated with Removal of Transcription Factors within the Promoter Region
Source: PLoS One. 2011 Nov 23;6(11):e28058. doi: 10.1371/journal.pone.0028058 (PMC3223225; doi:10.1371/journal.pone.0028058)
Supplement: Table S1 — List of primers used for 3′ UTR cloning and amplification. (DOC) [file pone.0028058.s010.doc]

Table S1:

| **Gene Name** | **Primer Name** | **Sequence (5'-3' direction)** | **Ta (°C)** |
| --- | --- | --- | --- |
| rpL32_8.1 | Forward cloning | TTTCAGCGGGGTGGTGTTC | 57 |
|  | Reverse cloning | CAGATACAATACAGCATTCATTCCAG | 57 |
|  | Forward 3'UTR specific | GTAGGCTGTTTTATCAGTATAATG | 57 |
|  | Reverse 3'UTR specific | GTAGGCTAATGAATGATGCGATAG | 57 |
| rpL32_9.1 | Forward cloning | AGCTGGCATCGTGGTCACC | 58 |
|  | Reverse cloning | AACTCGGGTCCGAACCATG | 58 |
|  | Forward 3'UTR specific | GTGCTTATGGCTGGATTGTTCG | 59 |
|  | Reverse 3'UTR specific | GATTTAGTTGTATTCTCATAGAGG | 59 |
| rpL32_9.2 | Forward cloning | ACGAAGAAAGCCAAGGAGATTAGTG | 58 |
|  | Reverse cloning | CAAGGAAAGAAAAGTGACAGGGC | 58 |
|  | Forward 3'UTR specific | GGATGAGTAATTTTGGACTTGG | 58 |
|  | Reverse 3'UTR specific | CTTTCACGTCATAATTGCTATAC | 58 |
| rpL32_9.3 | Forward cloning | CAATGTCTCAACGAAGAAGCG | 57 |
|  | Reverse cloning | ACGTCCTCGACGATCACAAGAG | 57 |
|  | Forward 3'UTR specific | TTGTTACGCTGGTTGAGCTG | 58 |
|  | Reverse 3'UTR specific | GTAGAACTATAGTGCCCAAAG | 58 |
