# Supplementary material for: Transcriptional Downregulation of Rice rpL32 Gene under Abiotic Stress Is Associated with Removal of Transcription Factors within the Promoter Region
Source: PLoS One. 2011 Nov 23;6(11):e28058. doi: 10.1371/journal.pone.0028058 (PMC3223225; doi:10.1371/journal.pone.0028058)
Supplement: Table S2 — List of primers used for promoter cloning of rpL32_8.1 gene. (DOC) [file pone.0028058.s011.doc]

Table S2:

| **Gene Name** | **PCR Round** | **Primer Name** | **Sequence (5'-3' direction)** | **Ta (°C)** |
| --- | --- | --- | --- | --- |
| rpL32_8.1 | 1 | Forward | GGGTTGCTACCAAAACTTAGC | 55 |
|  |  | Reverse | CACTATGGGGCCTCTTGAAC | 57 |
|  | 2 | Forward | ATGGTAGCAATCCAAATACAGC | 55 |
|  |  | reverse | ACCCTCTTCTTCACGATCTTC | 55 |
