# Supplementary material for: Transcriptional Downregulation of Rice rpL32 Gene under Abiotic Stress Is Associated with Removal of Transcription Factors within the Promoter Region
Source: PLoS One. 2011 Nov 23;6(11):e28058. doi: 10.1371/journal.pone.0028058 (PMC3223225; doi:10.1371/journal.pone.0028058)
Supplement: Table S3 — List of primers used for generating promoter deletions of rpL32_8.1 gene. (DOC) [file pone.0028058.s012.doc]

| **Gene Name** | **Primer Name** | **Sequence (5'-3' direction)** | **Ta (°C)** |
| --- | --- | --- | --- |
| rpL32_8.1 | Reverse (R1) | CCGGATCCCTCTGCTCCACCAAAACAC | 55 |
|  |  | __BamHI |  |
|  | DF0 forward | GGCTGCAGATGGTAGCAATCCAAATACAG | 54 |
|  | DF1 forward | GGCTGCAGCAATGGATCAGAGAAAAGGGT | 55 |
|  | DF2 forward | GGCTGCAGTGTTGTTCAAAGTGAAACAAC | 52 |
|  | DF3 forward | GGCTGCAGAAAAATCCAAACGGCAACAGAC | 55 |
|  | DF4 forward | GGCTGCAGTTCCATCTCACGAGATCCTA | 55 |
|  |  | ___PstI |  |

Table S3:
