# Supplementary material for: Transcriptional Downregulation of Rice rpL32 Gene under Abiotic Stress Is Associated with Removal of Transcription Factors within the Promoter Region
Source: PLoS One. 2011 Nov 23;6(11):e28058. doi: 10.1371/journal.pone.0028058 (PMC3223225; doi:10.1371/journal.pone.0028058)
Supplement: Table S4 — List of primers used for semiquantitative RT-PCR analysis. (DOC) [file pone.0028058.s013.doc]

Table S4:

| **Gene name** | **Primer name** | **Primer sequence (5'-3' direction)** | **Ta (^o^C)** |
| --- | --- | --- | --- |
| rpL32_8.1 | Forward | CTCCGATCCGAAGAGATGG | 57 |
|  | Reverse | CGCGACAGTTTCCTACAATC | 57 |
| rpL32_9.1 | Forward | GCAGGAGTTTGCAAAGGTAAC | 56 |
|  | Reverse | ACGAACAATGCAGCCATAAG | 56 |
| rpL32_9.2 | Forward | CCTTCTCTCCTCCCGAACTC | 55 |
|  | Reverse | GCAGGCAAAAACCAAGTCC | 55 |
| rpL32_9.3 | Forward | AGGGGTTCGCGAAGGTAAC | 55 |
|  | Reverse | ACGCGACCAAAAAACAGC | 55 |
| EF-1α | Forward | TTTCACTCTTGGTGTGAAGCAGAT | 62 |
|  | Reverse | GACTTCCTTCACGATTTCATCGTAA | 62 |
