# Supplementary material for: Transcriptional Downregulation of Rice rpL32 Gene under Abiotic Stress Is Associated with Removal of Transcription Factors within the Promoter Region
Source: PLoS One. 2011 Nov 23;6(11):e28058. doi: 10.1371/journal.pone.0028058 (PMC3223225; doi:10.1371/journal.pone.0028058)
Supplement: Table S5 — List of primers used for in vivo DMS-LMCR footprinting. (DOC) [file pone.0028058.s014.doc]

Table S5:

| **Primer Name** | **Sequence (5-3' direction)** | **Ta (°C)** |
| --- | --- | --- |
| LINKER1 | GCGGTGACCCGGGAGATCTGAATTC |  |
| LINKER2 | GAATTCAGATC |  |
| R1 (for blunt end) | GAGGATGGCGAAGGGTTC | 58 |
| R2(for amplification) | CGACGGCTAGGATCTCGTGAG | 60 |
| R3 (for extension) | TCGTGAGATGGAAGCGGACG | 63 |
| F1(for blunt end) | AAAATCCAAACGGGAACAGAC | 59 |
| F2 (for amplification) | TGCTTGGACATCACGGGATCTCC | 62.5 |
| F3 (for extension) | TCACGAATCTCCGTATAGTTTGGGC | 64 |
